# Presence and Role of the Type 3 Fimbria in the Adherence Capacity of *Enterobacter hormaechei* subsp. *hoffmannii*

**DOI:** 10.3390/microorganisms12071441

**Published:** 2024-07-16

**Authors:** Valentina Fernández-Yáñez, Valentina Ibaceta, Alexia Torres, Roberto M. Vidal, Isidora Schneider, Valeria Schilling, Cecilia Toro, Carolina Arellano, Paola Scavone, Ignacio Muñoz, Felipe Del Canto

**Affiliations:** 1 Departamento de Biología, Facultad de Química y Biología, Universidad de Santiago de Chile, Av. Libertador Bernardo O’Higgins 3363, Santiago 9170022, Chile; valentina.fernandezy@usach.cl; 2 Programa de Microbiología y Micología, Instituto de Ciencias Biomédicas, Facultad de Medicina, Universidad de Chile, Av. Independencia 1027, Independencia, Santiago 8380453, Chile; 3 Instituto Milenio de Inmunología e Inmunoterapia, Facultad de Medicina, Universidad de Chile, Av. Independencia 1027, Independencia, Santiago 8380453, Chile; 4 Departamento de Bioquímica y Biología Molecular, Facultad de Ciencias Químicas y Farmacéuticas, Universidad de Chile, Santiago 8380453, Chile; 5 Laboratorio de Biofilms Microbianos, Departamento de Microbiología, Instituto de Investigaciones Biológicas Clemente Estable, Montevideo 11600, Uruguay

**Keywords:** *Enterobacter hormaechei* subsp. *hoffmannii*, type 3 fimbria, bacterial adherence

## Abstract

*Enterobacter hormaechei*, one of the species within the *Enterobacter cloacae* complex, is a relevant agent of healthcare-associated infections. In addition, it has gained relevance because isolates have shown the capacity to resist several antibiotics, particularly carbapenems. However, knowledge regarding colonization and virulence mechanisms of *E. hormaechei* has not progressed to the same extent as other E*nterobacteriaceae* species as *Escherichia coli* or *Klebsiella pneumoniae*. Here, we describe the presence and role of the type 3 fimbria, a chaperone-usher assembled fimbria, which was first described in *Klebsiella* spp., and which has been detected in other representatives of the *Enterobacteriaceae* family. Eight Chilean *E. cloacae* isolates were examined, and among them, four *E. hormaechei* isolates were found to produce the type 3 fimbria. These isolates were identified as *E. hormaechei* subsp. *hoffmannii*, one of the five subspecies known. A mutant *E. hormaechei* subsp. *hoffmannii* strain lacking the *mrkA* gene, encoding the major structural subunit, displayed a significantly reduced adherence capacity to a plastic surface and to Caco-2 cells, compared to the wild-type strain. This phenotype of reduced adherence capacity was not observed in the mutant strains complemented with the *mrkA* gene under the control of an inducible promoter. Therefore, these data suggest a role of the type 3 fimbria in the adherence capacity of *E. hormaechei* subsp. *hoffmannii*. A screening in *E. hormaechei* genomes contained in the NCBI RefSeq Assembly database indicated that the overall presence of the type 3 fimbria is uncommon (5.94–7.37%), although genes encoding the structure were detected in representatives of the five *E. hormaechei* subspecies. Exploration of complete genomes indicates that, in most of the cases, the *mrkABCDF* locus, encoding the type 3 fimbria, is located in plasmids. Furthermore, sequence types currently found in healthcare-associated infections were found to harbor genes encoding the type 3 fimbria, mainly ST145, ST78, ST118, ST168, ST66, ST93, and ST171. Thus, although the type 3 fimbria is not widespread among the species, it might be a determinant of fitness for a subset of *E. hormaechei* representatives.

## 1. Introduction

*Enterobacter hormaechei* is a Gram-negative bacterium belonging to the *Enterobacteriaceae* family and one of the most common representatives of the *Enterobacter cloacae complex* (ECC) [1]. *E. hormaechei* is a frequent agent of diverse infections in humans, mainly opportunistic infections in healthcare-associated settings, including urinary tract infections and bloodstream infections, among others [1,2,3]. Five *E. hormaechei* subspecies have been established: *E. hormaechei* subsp. *hormaechei*, *E. hormaechei* subsp. *hoffmannii*, *E. hormaechei* subsp. *oharae*, *E. hormaechei* subsp. *steigerwaltii*, and *E. hormaechei* subsp. *xiangfangensis* [1,4]. Many aspects regarding knowledge about *E. hormaechei* biology are undergoing evolution. In fact, taxonomy is one of these aspects. A recent scheme for species distribution proposed *E. hormaechei* to be a single species, without a subspecies, leaving *E. hoffmannii* and *E. xiangfangensis* as separate species (not subspecies) [5]. Representatives of *E. hormaechei* subsp. *oharae* and *E. hormaechei* subsp. *steigerwaltii* would be part of the *E. xiangfangensis* species [5]. This scheme has not been incorporated yet by the List of Prokaryotic Names with Standing in Nomenclature [4], but the situation reflects that more research is required to gain insights into *E. hormaechei* life.

It is well established that *Enterobacter* species have developed resistance to multiple antibiotics, and this is the reason why they have been part of the ESKAPE and ESCAPE groups of bacteria [6,7]. In addition, carbapenem-resistant and third-generation cephalosporin-resistant *Enterobacter* species were considered within the critical group, along with other *Enterobacteriaceae* family members, in the Bacterial Priority Pathogens List recently published by the World Health Organization [8]. However, the emerging status of *Enterobacter* species, and particularly *E. hormaechei*, has not been accompanied by progress in knowledge of the colonization and infectious mechanisms, at least to a similar extent as other relative species such as *Klebsiella pneumoniae* and *Escherichia coli*. Therefore, gaining insights into the colonization and virulence determinants of *E. hormaechei* is required, in parallel to surveilling the antibiotic resistance status, because evolution might be followed up, and alternative therapies could be designed [9].

The type 3 fimbria is an adherence structure assembled by the chaperone–usher pathway, first discovered in *Klebsiella* and found widely distributed among the *K. pneumoniae* species [10,11]. The type 3 fimbria is composed of the major structural subunit MrkA, the minor subunit MrkF, and the tip subunit MrkD, which would act as the adhesin [12]. MrkB is the chaperone that assists structural subunit assembly at the usher MrkC, an outer membrane porin-like protein [12]. The type 3 fimbria system has been relatively well studied in *K. pneumoniae*; in fact, the MrkA protein has been proposed as a target for anti-adherence therapies [13,14]. Besides *K. pneumoniae*, type 3 fimbria can be produced by other representatives of the *Enterobacteriaceae* family, such as *Klebsiella oxytoca*, *E. coli*, *Citrobacter freundii*, and *Citrobacter koseri* [15]. In addition, the presence of type 3 fimbria has been detected in some *Enterobacter* species, and the *mrkB* gene has been found in *E. hormaechei* subsp. *oharae* [16,17,18], but its actual contribution to their adherence capacity has not been addressed. In this study, we report the evaluation of the role of type 3 fimbria in the adherence capacity of an *E. hormaechei* subsp. *hoffmannii* clinical isolate obtained in Chile and the presence of the locus encoding the structure among *E. hormaechei* genomes.

## 2. Materials and Methods

Strains: A group of eight strains belonging to the *E. cloacae* complex (Eclo1-UCH, Eclo5-UCH, Eclo6-UCH, Eh12-UCH, Eh13-UCH, Eh18-UCH, Eclo29-UCH, and Eh31-UCH), previously isolated in Santiago (Chile) during the SENTRY antimicrobial resistance study [19], were initially selected for this work (Table 1). Four of these strains (Eh12-UCH, Eh13-UCH, Eh18-UCH, and Eh31-UCH) were positive for the detection of the *mrkA* gene by PCR using primers mrkA-F and mrkA-R (primers listed in Table S1). PCR mixes comprised 1X reaction buffer, 1.5 mM MgCl_2_, 0.8 mM dNTPs, 10 pmol primers, and 0.6 U Taq DNA polymerase (GoTaq^®^ G2, Promega, Madison, WI, USA). Amplification conditions were (1) one cycle at 94 °C for 5 min; (2) 30 cycles including denaturation at 94 °C for 30 s, annealing at 57 °C for 30 s, and extension at 72 °C for 30 s; (3) a final extension cycle at 72 °C for 10 min. Later, the four isolates were identified as *E. hormaechei* subsp. *hoffmannii* after genome sequencing (see “Genome/bioinformatical analyses” below).

Antimicrobial resistance profile: Susceptibility to common antibiotics was tested by the Kirby–Bauer method, according to the guidelines of the Clinical and Laboratory Standard Institute (CLSI) [22]. Antibiotics tested were amikacin (30 μg), cefepime (30 μg), cefotaxime (30 μg), cefotaxime–clavulanic acid (30/10 μg), cephalothin (30 μg), chloramphenicol (30 μg), kanamycin (30 μg), levofloxacin (10 μg), streptomycin (10 μg), tetracycline (30 μg), trimethoprim (5 μg) (all obtained from Oxoid, Thermo Fisher, Waltham, MA, USA); ampicillin (10 μg) and gentamicin (10 μg) (both obtained from BD, Franklin Lakes, NJ, USA), ciprofloxacin (5 μg), imipenem (10 μg), meropenem (10 μg), nalidixic acid (30 μg), nitrofurantoin (300 μg), piperacillin–tazobactam (100/10 μg), and trimethoprim–sulfamethoxazole (1.25/23.75 μg) (all obtained from Mast Group, Bootle, UK). *E. coli* strain ATCC 25922 was used as a control.

Genome/bioinformatical analyses: Draft genomic sequences were obtained for strains Eh12-UCH, Eh13-UCH, Eh18-UCH, and Eh31-UCH, as they were recognized as positive for *mrkA*. Briefly, strains were grown in lysogeny broth (LB, Lennox formula, Thermo Fisher Scientific, Waltham, MA, USA), and genomic DNA was purified using the Wizard Genomic DNA Purification kit (Promega, Madison, WI, USA). The integrity of the product was determined by electrophoresis in 1% agarose gel and ethidium bromide staining. Sequencing was performed at MicrobesNG (Birmingham, UK) using the Illumina MiSeq platform (Illumina, San Diego, CA, USA). Draft genomes were provided after assembly with SPAdes 3.14 [23]. Assembly metrics (Genome length and N50) were obtained by using QUAST v5.0.2 [24], while percentages of completeness and contamination were obtained with CheckM v1.2.2 [25]. The species were identified using “Identify Species” (available at https://pubmlst.org/species-id, accessed on 27 April 2024) [26]. Subspecies were identified by performing a phylogenetic analysis with kSNP 3.1 [27], including genomes of strains *E. hormaechei* subsp. *hoffmannii* DSM 14563, *E. hormaechei* subsp. *hormaechei* ATCC 49162, *E. hormaechei* subsp. *oharae* DSM 16687, *E. hormaechei* subsp. *steigerwaltii* DSM 16691, and *E. hormaechei* subsp. *xiangfangensis* LMG 27195 as controls (Table S2) [5]. The main features of the sequences obtained are shown in Table 2. The tree image was obtained from the Interactive Tree of Life (iToL) server. In addition, subspecies were established by determining the average nucleotide identity (ANI) using the FastANI software v1.1 [28]. The subspecies for which the highest ANI value was obtained, being this ≥96%, was considered as the subspecies of the interrogated genome. Sequence types were identified using the *E. cloacae* scheme in the in silico multiple locus sequence typing software mlst 2.18 [29,30]. Furthermore, the results of determining the antibiotic resistance profiles were complemented by detecting antibiotic resistance genes and their associated profiles using ResFinder 4.5.0 [31,32].

Screening of the type 3 fimbria encoding locus *mrkABCDF*, and the individual genes, was performed using the Large-Scale Blast Score Ratio (LS-BSR) software with the blastn or the tblastn options (available at https://github.com/jasonsahl/LS-BSR, accessed on 1 May 2024). Sequences were selected from the *K. pneumoniae* Kp13 strain’s genome (Table S2) [33]. The genomes screened included the Chilean isolates and *E. hormaechei* genomes obtained from NCBI Assembly RefSeq database (https://www.ncbi.nlm.nih.gov/genbank/, accessed on 1 May 2024) [34]. Records with BSR ≥ 0.9 were considered positive, according to a previous report of our group and others [35,36,37]. Template sequences selected for screening and their accession codes are shown in Table S2. The presence of the *mrkABCDF* locus in chromosomes or plasmids was explored in annotations (genomic files in .gbff extension) of fully sequenced genomes contained in the NCBI Assembly RefSeq database. Subspecies were determined by performing the ANI analysis, and sequence types were determined by using the mlst software, as indicated above.

Allelic exchange and complementation: The *mrkA*, which encodes the type 3 fimbria major structural subunit, was knocked out in Eh13-UCH by the allelic exchange procedure, according to the protocol described by Sharan et al. [21]. Briefly, the strain was transformed with approximately 100 ng of the purified pSIM9 plasmid by electroporation at 1700 V and recombinants were selected in LB agar plates containing 12.5 μg/mL chloramphenicol (Sigma Aldrich, St Louis, MO, USA) at 30 °C. Although the strain was resistant to disks containing 10 μg/mL of ampicillin, it was sensitive to 100 μg/mL in LB (Sigma Aldrich, St Louis, MO, USA). The next day, chloramphenicol-resistant colonies were transformed with approximately 500 ng of a purified linear DNA fragment containing the kanamycin resistance gene *aph* flanked by 40 nt sequences identical to both *mrkA* ends. This product was generated by PCR using primers *mrkA*-mutF and *mrkA*-mutR, described in Table S1, and the plasmid pCLF4 as template. PCR conditions were (1) initial denaturation at 94 °C for 5 min; (2) 30 cycles including denaturation at 94 °C for 30 s, annealing at 56 °C for 30 s, and elongation at 72 °C for 1 min; (3) final elongation at 72 °C for 10 min. Electroporation was performed at 17,000 V and recombinant strains were selected by seeding the bacterial suspension in LB plates containing kanamycin (50 μg/mL, Sigma Aldrich, St Louis, MO, USA). Verification of the allelic exchange was performed by PCR from colony lysates and then from purified DNA using primers mrkA-Nde and K1 (Table S1). PCR conditions were (1) initial denaturation at 94 °C for 5 min; (2) 30 cycles including denaturation at 94 °C for 30 s, annealing at 56 °C for 30 s, and elongation at 72 °C for 30 s; (3) final elongation at 72 °C for 10 min.

Complementation of the Eh13-UCH*mrkA* strain was performed by transformation with the pVB1 expression plasmid harboring the *mrkA* gene. For this purpose, the *mrkA* gene was amplified by PCR from Eh13-UCH purified genomic DNA using primers mrkA-nde and mrkA-bam. The product was purified, digested with restriction endonucleases NdeI (FastDigest, Thermo Fisher Scientific, Waltham, MA, USA) and BamHI (FastDigest, Thermo Fisher Scientific, Waltham, MA, USA) and then ligated into the pVB1 plasmid, previously digested with the same enzymes and purified. *E. coli* DH5α was electroporated with the ligation mix and clones harboring the plasmid were selected by seeding onto LB agar plates containing ampicillin (100 μg/mL). Presence of recombinant plasmids was checked by colony PCR using primers pVB1-F and mrkA-bam. Recombinant plasmid was purified and checked again by PCR and by digestion with NdeI and BamHI endonucleases. Finally, the purified recombinant plasmid was introduced in Eh13-UCHΔ*mrkA* by electroporation at 1700 V and selection of clones in LB agar plates containing ampicillin (100 μg/mL). Expression of the *mrkA* gene in the complemented Eh13-UCH*mrkA*/*mrkA* was induced by adding 2 mM of m-tuolic acid (Sigma Aldrich, St Louis, MO, USA) to the culture media.

Western blot: The presence of the type 3 fimbriae was established by detecting MrkA by Western blot in heat-extracted proteins. Briefly, bacteria were cultured in 10 mL of LB broth, containing supplements if necessary (antibiotics kanamycin, ampicillin, or m-tuolic acid if necessary). After overnight incubation at 37 °C, without shaking, tubes were centrifuged at 3000× *g* for 10 min at room temperature (RT). The sediment was gently suspended in 1 mL of phosphate buffer saline (PBS, Merck Millipore, Burlington, MA, USA), centrifuged again under the same conditions, and suspended in 100 µL of PBS. The suspension was heated at 60 °C for 30 min and then centrifuged at 3000× *g* for 10 min at RT. The supernatant was recovered, centrifuged again under the same conditions, and the supernatant was recovered again. This fraction represents the heat-extracted proteins that have been shown to contain fimbrial major structural subunits [38]. The concentration of proteins was determined by the Bradford method [39]. A volume containing 1 µg of heat-extracted proteins was subjected to sodium dodecyl sulfate–polyacrylamide gel electrophoresis (SDS-PAGE) under denaturing conditions in a two phases polyacrylamide gel (4% concentrating phase and 15% separating phase) for 90 min at 100 V. The separated proteins were transferred to a nitrocellulose membrane in a wet transference apparatus (Mini Trans-Blot, BioRad, Hercules, CA, USA), and then, the protein binding sites were blocked with 1% bovine serum albumin (BSA, Merck Millipore, Burlington, MA, USA) dissolved in Tris-buffered saline solution containing 0.05% Tween 20 (TBS-T, Merck Millipore, Burlington, MA, USA). The membrane was incubated with a polyclonal rabbit anti-MrkA antibody (Poly Express custom antibody production, Genscript, Piscataway, NY, USA) in a 1:1000 dilution for 1 h at RT. After three washes with TBS-T buffer, 10 min at RT each, the membrane was incubated with a secondary goat anti-rabbit IgG conjugated to alkaline phosphatase (Thermo Fisher Scientific, Waltham, MA, USA) in a 1:5000 dilution for 1 h at RT. The membrane was washed three times with TBS-T and once with distilled water to finally reveal the presence of immunoreactive bands by adding a mix of the chromogenic alkaline phosphatase substrates nitro blue tetrazolium chloride (NBT) and 5-bromo-4-chloro-3-indolyl-phosphate (BCIP) (Novex, Thermo Fisher Scientific, Waltham MA, USA).

Immunogold staining: The presence of the type 3 fimbria was also established in Eh13-UCH, and its derivative strains, by detecting the MrkA protein by immunogold staining over whole non-permeabilized bacteria. An aliquot of 1 mL of an overnight culture in LB, grown at 37 °C without shaking and containing supplements, if necessary (antibiotics and/or m-tuolic acid), was concentrated by centrifugation at 3000× *g* and gently suspended in 100 μL of LB. Ten microliters of this suspension was deposited over 200-mesh formvar/carbon nickel grids (Electron Microscopy Sciences, Hatfield, PA, USA) and incubated for 20 min at 37 °C inside a humidified chamber. After three washes with PBS, grids were incubated with the blocking solution, 1% BSA 0.01 M glycine in PBS, for 1 h at RT (Glycine obtained from Merck Millipore, Burlington, MA, USA). Three washes were then performed with washing solution, 1% BSA 0.05% Tween 20 in PBS (PBS-BT), and the grids were incubated with the primary antibody anti-MrkA in a 1:10 dilution, dissolved in PBS-BT, for 1 h at RT. Three washes with PBS-BT were carried out, and the grids were incubated with the secondary antibody, anti-rabbit IgG conjugated with 10 nm gold particles (Sigma Aldrich, St Louis, MO, USA) in a 1:10 dilution for 1 h at RT. After three washes with PBS-BT, bacteria attached to the grids were fixed with 2% glutaraldehyde (Merck Millipore, Burlington, MA, USA) for 10 min at RT and then washed three times with ultrapure water. Finally, grids were incubated with 0.5% phosphotungstic acid (Merck Millipore, Burlington, MA, USA) for 30 s, washed three times with ultrapure water, and allowed to dry for 1 h at 37 °C. Bacteria were visualized in a Hitachi HT7700 transmission electron microscope (Minato-ku, Tokyo, Japan) at 80 kV at the Center for the Development of Nanoscience and Nanotechnology, Universidad de Santiago de Chile.

Adherence assays: The role of the type 3 fimbria was established by assessing the adherence capacity of Eh13-UCH and its derivative strains over empty polystyrene cell culture plates and over Caco-2 cells. In the first case, 96 black well plates with optical bottoms were used (Thermo Fisher Scientific 165305, Waltham, MA, USA). Approximately 10^6^ colony forming units (CFU) of each strain were put in each well in a final volume of 100 µL of LB or Dulbecco’s modified Eagle medium (DMEM) containing 4.5 mg/mL glucose, sodium pyruvate, and glutamine (Thermo Fisher Scientific, Waltham, MA, USA). Antibiotics kanamycin, ampicillin, and/or the inducer m-tuolic acid were included for those wells receiving the mutant Eh13-UCHmrkA strain and strains harboring the pBV1 plasmid when necessary. The plate was incubated at 37 °C for 3 h or 48 h. After the incubation time was completed, for the 3 h assay, wells were washed three times with PBS, and attached bacteria were stained with 4′,6-diamidino-2-phenylindole (DAPI, Merck Millipore, Burlington, MA, USA) in a concentration of 1 µg/mL for 1 min. Then, three washes with distilled water were performed, and fluorescence intensity was measured from the bottom in a Synergy HT multi-plate reader (BioTek Instruments, Winooski, VT, USA). For the 48 h assay, wells were washed three times with PBS after the incubation time was completed and attached bacteria were fixed with 99% methanol (Merck Millipore, Burlington, MA, USA) for 15 min at RT. Wells were washed three times with distilled water and bacteria were stained with 0.1% crystal violet (Merck Millipore, Burlington, MA, USA) for 30 min at RT. After three washes with distilled water, 100 µL of a mix of ethanol/acetone (80%/20%) (Merck Millipore, Burlington, MA, USA) was added to each well. This volume was recovered and optical density (OD) at 595 nm was measured.

Confluent layers of Caco-2 cells were used for cell adherence assays. This model has been previously tested with *E. hormaechei* [40]. Cells were maintained in DMEM high glucose supplemented with 10% fetal bovine serum (Cytiva, Amersham, UK) and 1% antibiotic/antimycotic mix (Thermo Fisher Scientific, Waltham, MA, USA), at 37 °C in an atmosphere containing 95% air/5% CO_2_. Approximately 1.9 × 10^4^ cells were seeded onto 24-well plates (Nest, Wuxi, Jiangsu, China) and kept until reaching confluence. Before the infection, cells were washed once with PBS and then infected at a multiplicity of infection (MOI) of 10 bacteria per cell. Infection lasted for 30 min or 3 h at 37 °C under the 95% air/5% CO_2_ atmosphere. After five washes with PBS, cells were lysed with 0.1% Triton X-100 (Winkler Ltd., Santiago, Chile), and cell-associated viable bacteria were quantified by performing serial dilutions and seeding drops onto LB plates.

Statistical analyses: Data were analyzed with the Brown–Forsythe and Welch’s ANOVA tests followed by Dunnet’s T3 multiple comparison test, using the GraphPad Prism v9 software. Significant differences were established when *p* < 0.05.

## 3. Results

### 3.1. Production of Type 3 Fimbria by E. hormaechei subsp. hoffmannii

**Figure 1:**
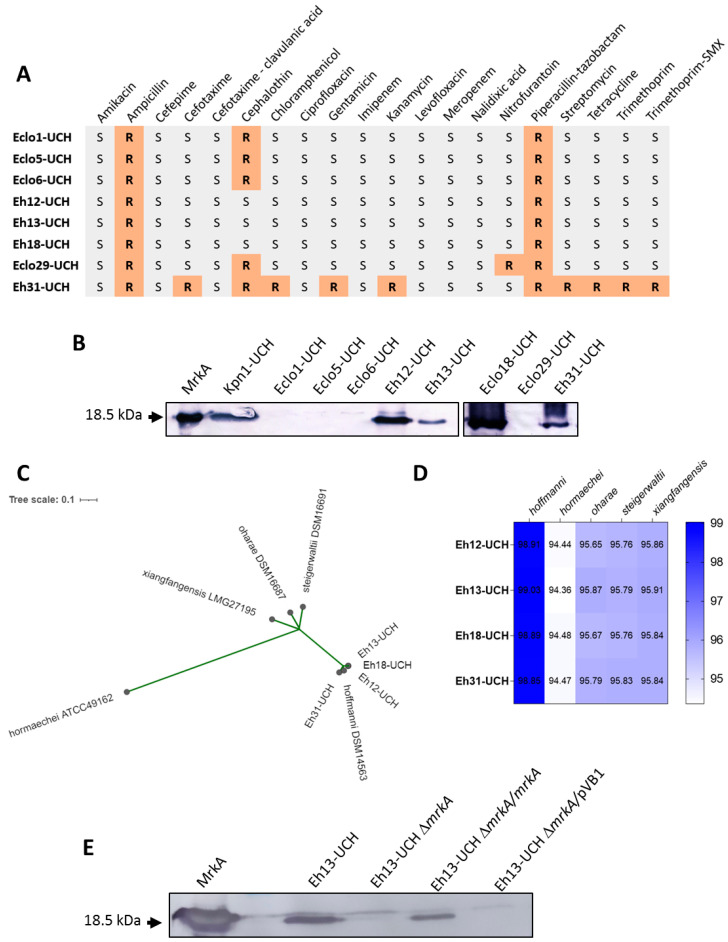
Presence of the type 3 fimbriae among *Enterobacter cloacae* complex strains and *E. hormaechei* subsp. *hoffmannii* strains: (**A**) Antibiotic susceptibility profile of eight *E. cloacae* complex strains. S: susceptible, R: resistant. Trimethropim-SMX: trimethoprim–sulfamethoxazole. (**B**) The type 3 fimbria major structural subunit MrkA was detected by Western blot in heat-extracted proteins. The purified mature MrkA protein and extracts obtained from the *Klebsiella pneumoniae* strain Kpn1-UCH were used as positive controls. (**C**) Maximum parsimony phylogenetic tree to identify *E. hormaechei* subspecies. Genomes of strains Eh12-UCH, Eh13-UCH, Eh18-UCH, and Eh31-UCH were included along with genomes of the type strains *E. hormaechei* subsp. *hoffmannii* DSM 14563, *E. hormaechei* subsp. *hormaechei* ATCC 49162, *E. hormaechei* subsp. *oharae* DSM 16687, *E. hormaechei* subsp. *steigerwaltii* DSM 16691, and *E. hormaechei* subsp. *xiangfangensis* LMG 27195 [5]. The tree was built based on 48,321 core SNPs. (**D**) Identification of *E. hormaechei* subspecies by average nucleotide identity (ANI) analysis. The heat map represents the results for the same set of genomes included in (**C**). (**E**) Detection of MrkA by Western blot in heat-extracted proteins obtained from the mutant strain Eh13-UCHΔ*mrkA* and its derivative complemented strains.

Eight clinical isolates identified as *Enterobacter cloacae* (Table 1) were included in a screening of loci encoding fimbriae assembled by the chaperone–usher pathway. Disk susceptibility test indicated that seven of them were sensitive to most of the antibiotics tested (Figure 1A). All the strains were resistant to ampicillin and piperacillin–tazobactam, while five were resistant to cephalothin. Only one strain, Eh31-UCH, showed resistance to multiple antibiotics, including resistance to beta-lactam drugs apparently determined by an extended spectrum β-lactamase (ESBL), as clavulanic acid inhibited cefotaxime resistance. No resistance to carbapenems was detected. Four of the strains, Eh12-UCH, Eh13-UCH, Eh18-UCH, and Eh31-UCH, were positive for the detection of the *mrkA* gene, encoding the major structural subunit of the type 3 fimbriae, by PCR. This result was further confirmed by the detection of MrkA protein in heat-extracted proteins by Western blot (Figure 1B). The four strains that resulted negative in the detection of *mrkA* by PCR were also negative in the detection of MrkA by Western blot (Figure 1B). Sequencing of the positive strain’s genomes allowed identification as *Enterobacter hormaechei*. Multiple locus sequence type analysis indicated that Eh12-UCH, Eh13-UCH, and Eh18-UCH belong to the sequence type ST145 and Eh31-UCH to sequence type ST118 (Table 2). A phylogenetic analysis was conducted to identify the isolates using the *E. hormaechei* subspecies scheme [1,4,5]. Thus, genomes of type strains representing *E. hormaechei* subsp. *hoffmannii*, *E. hormaechei* subsp. *hormaechei*, *E. hormaechei* subsp. *oharae*, *E. hormaechei* subsp. *steigerwaltii*, and *E. hormaechei* subsp. *xiangfangensis* were included in a core genome SNP-based parsimony tree. The result indicated that the four isolates are *E. hormaechei* subsp. *hoffmannii* (Figure 1C). This was also proved by the determination of the ANI among the four Chilean isolates’ genomes and the reference genomes (Figure 1D). Furthermore, the prediction of the presence of antibiotic-resistance genes was consistent with the resistance phenotypes observed for Eh12-UCH, Eh13-UCH, Eh18-UCH, and Eh31-UCH. Thus, the presence of *bla*_ACT-14_ might explain the resistance of Eh12-UCH, Eh13-UCH, and Eh18-UCH to ampicillin and piperacillin–tazobactam (Table S3). On the other hand, ten different resistance genes were found in the Eh31-UCH genome, which explains the observed multi-resistant phenotype. Noteworthy is the presence of two genes encoding ESBLs, *bla*_TEM-1B_ and *bla*_SHV-12_ (Table S3).

In order to determine the role of the type 3 fimbriae in the adherence capacity of *E. hormaechei* subsp. *hoffmannii*, we selected Eh13 as the representative and removed the *mrkA* gene by allelic exchange. The *mrkA* gene was then provided back by transformation with the recombinant pVB1-*mrkA* plasmid. The outcomes of these processes were checked by Western blot (Figure 1E). MrkA was detected in heat-extracted proteins obtained from the wild-type Eh13-UCH strain and the complemented mutant Eh13-UCHΔ*mrkA*/*mrkA* strain, but it was not detected in extracts from the mutant Eh13-UCHΔ*mrkA* strain nor the mutant Eh13-UCHΔ*mrkA* harboring the empty pVB1 plasmid (Figure 1D).

**Figure 2:**
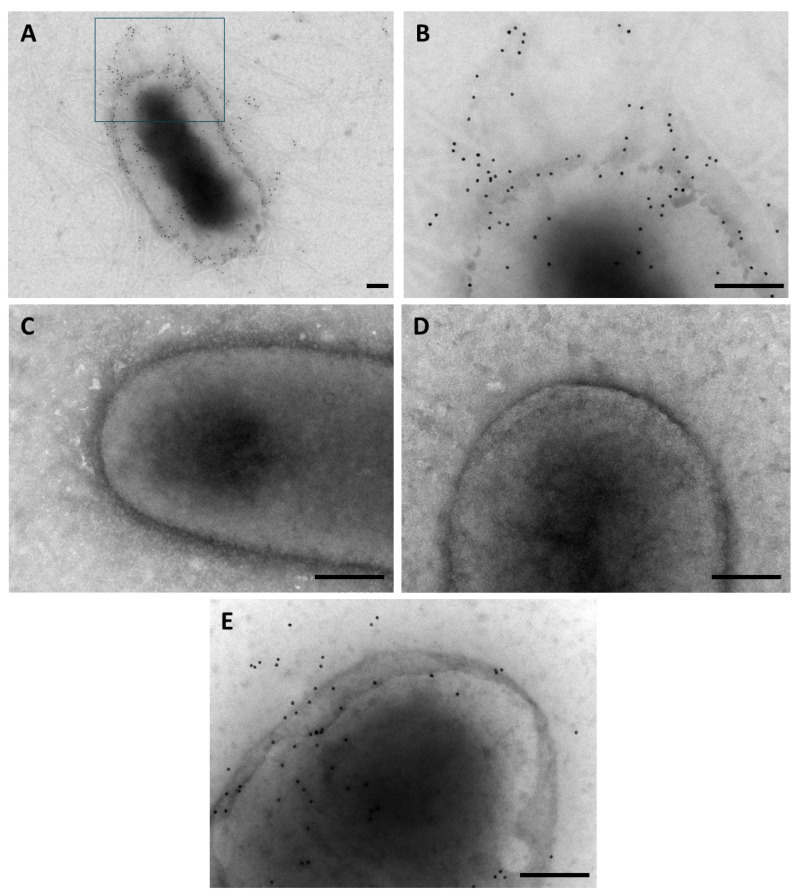
Detection of type 3 fimbria by immunogold staining. The presence of the structure was established using anti-MrkA and a secondary antibody conjugated with 10 nm gold particles over whole non-permeabilized bacteria: (**A**) Wild-type Eh13-UCH. (**B**) Higher magnification for the square depicted in (**A**) for wild-type Eh13-UCH. (**C**) Mutant Eh13-UCHΔ*mrkA*. (**D**) The mutant strain harboring the empty pVB1 plasmid (Eh13-UCHΔ*mrkA*/pVB1). (**E**) Complemented mutant strain (Eh13-UCHΔ*mrkA*/*mrkA*). Bars: 200 nm.

The presence of the type 3 fimbria was also established by immunogold staining in whole non-permeabilized bacteria. Gold particles were widely distributed along the entire surface of Eh13-UCH suggesting the presence of the type 3 fimbria (Figure 2A,B). In contrast, particles were not observed, or were very scarce, at the surface of the mutant strains Eh13-UCHΔ*mrkA* and Eh13-UCHΔ*mrkA* (Figure 2C,D), suggesting the absence of the structure. As expected, gold particles were evident in the complemented Eh13-UCHΔ*mrkA*/*mrkA* strain, indicating that the fimbria is present at the bacterial surface (Figure 2E).

### 3.2. Role of the Type 3 Fimbria in Adherence Capacity of E. hormaechei subsp. hoffmannii Eh13-UCH Strain

**Figure 3:**
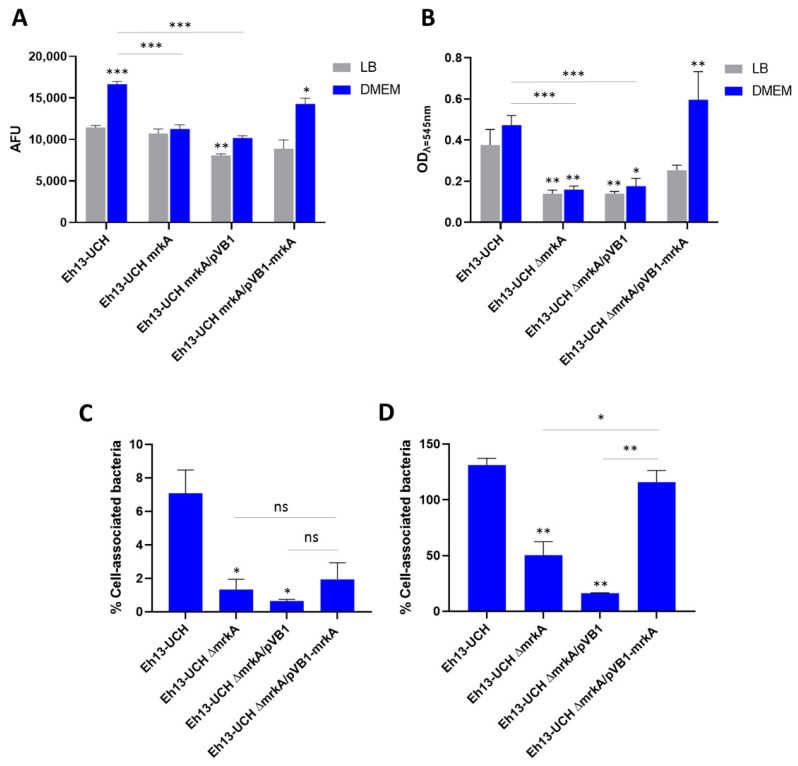
Evaluation of the adherence capacity of Eh13-UCH and its derivative strains: (**A**) Adherence over cell culture plates after 3 h of incubation, expressed as arbitrary fluorescence units (AFU). (**B**) Adherence over cell culture plates after 48 h of incubation, expressed as optical density measured at λ = 545 nm to detect crystal violet absorbance. LB: lysogeny broth. DMEM: Dulbecco’s modified Eagle medium. For (**A**,**B**), asterisks over the bars indicate significant differences compared to the level detected in the wild-type Eh13-UCH in LB. (**C**,**D**) Adherence capacity over Caco-2 cells after 30 min (**C**) or 3 h (**D**) of infection at a multiplicity of infection (MOI) of 10 bacteria/cell. Results are expressed as the percentage of cell-associated bacteria relative to the initial inoculum. For (**C**) and (**D**), asterisks over the bars indicate significant differences compared to the level detected in the wild-type Eh13-UCH. For all the cases, bars represent the mean ± the standard error. * *p* < 0.05, ** *p* < 0.01, *** *p* < 0.0001 according to Welch’s ANOVA test followed by Dunnett’s T3 multiple comparison test.

In order to evaluate the role of type 3 fimbria in the adherence capacity of *E. hormaechei* subsp. *hoffmanni*, adherence assays were performed with the Eh13-UCH strain and its derivatives over a plastic surface and Caco-2 cells. First, assays were carried out by incubating the strains over cell culture plates for 3 h. After removing the medium and repeatedly washing the wells, the attached bacteria were stained with DAPI, and fluorescence intensity was measured. Fluorescence levels were similar among all the strains tested when the assay was carried out in LB. The only significant difference was noticed with the mutant Eh13-UCHΔ*mrkA* strain harboring the empty pVB1 plasmid, which showed a lower adherence level than the wild type (Figure 3A). However, when the assay was performed in DMEM, a significant reduction in the adherence level was evident in both the mutant strain Eh13-UCHΔ*mrkA* and the mutant strain Eh13-UCHΔ*mrkA*/pVB1, compared to the wild-type’s level (Figure 3A). In fact, the fluorescence intensity was significantly higher in the wild-type EH13-UCH after incubation in DMEM, compared to the result obtained from the incubation in LB. In agreement with this observation, the complemented Eh13-UCHΔ*mrkA*/*mrkA* strain in DMEM showed an adherence level similar to that obtained with the wild type in DMEM (Figure 3A). Then, a similar assay was performed, but the incubation lasted for 48 h, and the presence of attached bacteria was measured by crystal violet staining. Regardless of the medium (LB or DMEM), results were consistent with the behavior observed in the 3 h assay performed in DMEM (Figure 3B). Thus, a significantly lower adherence capacity was observed in mutant strains Eh13-UCHΔ*mrkA* and the mutant strain Eh13-UCHΔ*mrkA*/pVB1, compared to the wild type, but this was not observed in the case of the complemented Eh13-UCHΔ*mrkA*/*mrkA* strain (Figure 3B).

In addition, infection assays were carried out over Caco-2 cells for 30 min or 3 h. Adherence capacity was expressed as the percentage of cell-associated bacteria, which means the percentage that represents the number of colony-forming units (CFU) recovered after lysis of the cell layer, relative to the initial inoculum. A significantly lower level of adherence was noticed in the mutant strains Eh13-UCHΔ*mrkA* and Eh13-UCHΔ*mrkA*/pVB1, compared to the wild type, at both times, 30 min and 3 h post-infection (Figure 3C,D). The difference was insignificant in the case of the complemented Eh13-UCHΔ*mrkA*/*mrkA* strain, although after 30 min of infection, the average percentage of cell-associated bacteria was approximately three times lower compared to the wild type (Figure 3C). In contrast, the percentage of cell-associated bacteria for Eh13-UCHΔ*mrkA*/*mrkA* after 3 h was similar to that observed for the wild type (Figure 3D).

### 3.3. Distribution of the mrkABCDF Locus among E. hormaechei

**Figure 4:**
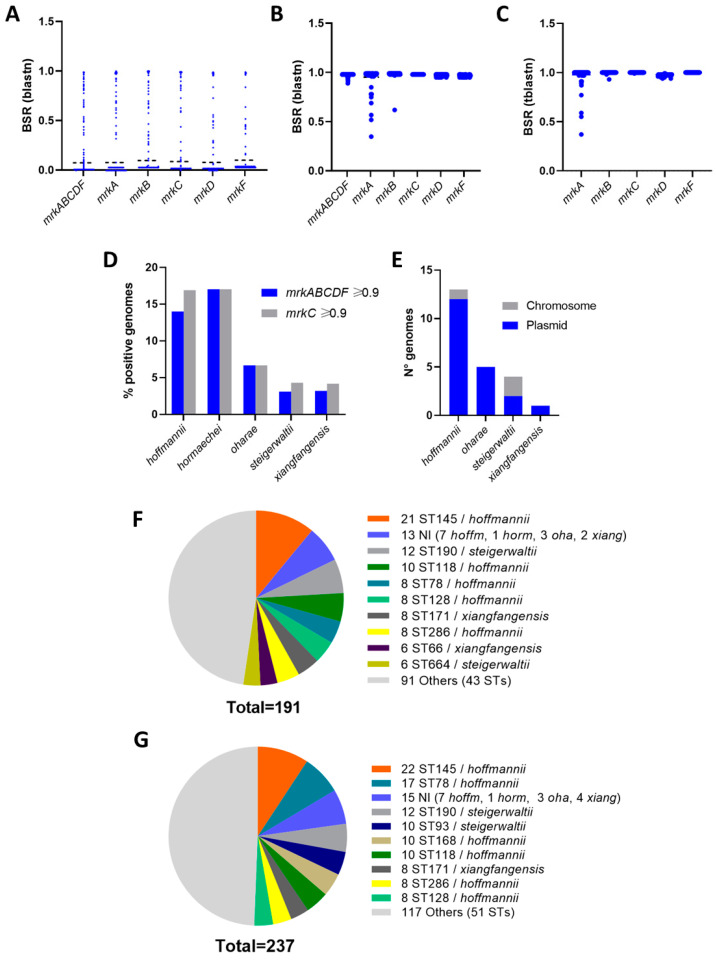
Distribution of the type 3 pili genes among *E. hormaechei* genomes contained in the NCBI Assembly RefSeq database: (**A**) Screening for the *mrkABCDF* locus and its individual genes using blastn. The graph shows the blast-score ratio (BSR) values. (**B**,**C**) Distribution of BSR values in the screening of individual genes among records that showed BSR equal to or higher than 0.9 for the screening of the *mrkABCDF* locus, using blastn (**B**) or tblastn (**C**). Horizontal dotted lines in (**A**–**C**) represent the means. (**D**) Identification of subspecies among positive records selected according to two criteria, BSR ≥ 0.9 for the *mrkABCDF* locus with blastn or BSR ≥ 0.9 for *mrkC* with tblastn. Numbers above the bars indicate the percentage of positive records among genomes representing each subspecies. Total numbers (100%) were 714 *E. hormaechei* subsp. *hoffmannii,* 47 *E. hormaechei* subsp. *hormaechei,* 165 *E. hormaechei* subsp. *oharae,* 1257 *E. hormaechei* subsp. *steigerwaltii*, and 1032 *E. hormaechei* subsp. *xiangfangensis.* (**E**) Localization of the *mrkABCDF* locus, analyzed in 23 complete genomes. No complete genomes representing *E. hormaechei* subsp. *hormaechei* were found. (**F**,**G**) Distribution of sequence type among *E. hormaechei* genome records positive for type 3 fimbriae, selected according to both criteria, BSR ≥ 0.9 in the screening for the *mrkABCDF* locus using blastn (**F**) or BSR ≥ 0.9 in the screening for *mrkC* with tblastn (**G**).

After obtaining evidence of the role of the type 3 fimbria in Eh13-UCH, we determined the distribution of the *mrkABCDF* locus among *E. hormaechei* genomes contained in the NCBI Assembly RefSeq database. Screening of 3215 genomes with blastn indicated that the presence of the *mrkABCDF* locus was uncommon, with only 191 genomes (5.94%) obtaining blast-score ratios (BSR) equal to or higher than 0.9 (Figure 4A,B). A similar distribution was observed for the individual genes, with 197 (6.12%), 208 (6.46%), 214 (6.66%), 214 (6.66%), and 223 (6.93%) genomes showing BSR ≥ 0.9 for *mrkA*, *mrkB*, *mrkC*, *mrkD*, and *mrkF*, respectively (Figure 4A,B). Screening of the individual genes using tblastn showed slightly higher BSRs, and the numbers of records with BSR ≥ 0.9 were 213 (6.63%), 230 (7.15%), 237 (7.37%), 214 (6.65%), and 223 (6.93%), for *mrkA*, *mrkB*, *mrkC*, *mrkD*, and *mrkF*, respectively (Figure 4C). Regardless of the algorithm used for the screening, blastn or tblastn, the higher degree of variation among records with BSR ≥ 0.9 for the *mrkABCDF* locus was found in the *mrkA* gene, which encodes the major structural subunit (Figure 4B,C).

We selected sets of genomes as type 3 pili positives for further analyses according to two criteria. The first group included 191 genomes showing BSR ≥ 0.9 for the *mrkABCDF* locus screening using blastn, and the second group included 237 genomes showing BSR ≥ 0.9 for the screening of *mrkC*, using tblastn. Thus, we identified the subspecies within both groups of genomes, positives for the type 3 pili genes, according to the ANI analysis. ANI values ranged from 98.10% to 99.95% for this identification. We considered the five subspecies recognized in the List of Prokaryotic names with Standing in Nomenclature [1,4]. Positive genomes were identified in the five cases, with the highest percentages obtained for *E. hormaechei* subsp. *hoffmannii* (14–17%, among 714 genomes) and *E. hormaechei* subsp. *hormaechei* (17%, among 47 genomes) (Figure 4D). Then, we explored annotations in a subset of 23 complete genomes to establish if the *mrkABCDF* locus was located on the chromosome or in any other extra-chromosome element. We found that the locus was located in plasmids, in most of the cases, regardless of the subspecies (Figure 4E). Regretfully, no *E. hormaechei* subsp. *hormaechei* complete genomes were found in the NCBI Assembly RefSeq database to be included in the analysis.

Finally, we determined the positive genomes’ sequence type according to the *E. cloacae* MLST scheme [29,30]. We found 52 different sequence types among genomes with BSR ≥ 0.9 for the screening of the *mrkABCF* locus and 60 sequence types among genomes with BSR ≥ 0.9 for the screening of the *mrkC* gene. Most of the positive genomes belonged to the ST145 sequence type, all identified as *E. hormaechei* subsp. *hoffmannii* (Figure 4F). Four other sequence types of this subspecies were also found among the most frequent, ST118, ST78, ST28, and ST286 (Figure 4F). In the set selected according to the *mrkC* BSR ≥ 0.9 criterion, ST168-genomes were also found as representatives of *E. hormaechei* subsp. *hoffmannii* (Figure 4G). Sequence types of *E. hormaechei* subsp. *steigerwaltii* and *E. hormaechei* subsp. *xiangfangensis* were also found among the most common, including ST93, ST190, and ST664, and ST66, and ST171, respectively (Figure 4F,G).

## 4. Discussion

In this work, we have described the presence and the role of type 3 fimbria in the adherence capacity of *E. hormaechei* subsp. *hoffmannii*. The presence was evidenced by the detection of the major subunit MrkA in heat-extracted proteins and also at the surface of whole bacteria in a representative strain isolated in Chile, the Eh13-UCH strain. This strain was identified as *E. hormaechei* subsp. *hoffmannii,* according to its draft genomic sequence, compared with type strains representing four *E. hormaechei* subspecies, *E. hormaechei* subsp. *hoffmanni*, *E. hormaechei* subsp. *hormaechei*, *E. hormaechei* subsp. *oharae*, *E. hormaechei* subsp. *steigerwaltii*, and *E. hormaechei* subsp. *xiangfangensis* [1,4].

Research to find and characterize colonization and virulence factors of *E. hormaechei,* and even in representatives of the *Enterobacter cloacae* complex, has not been carried out to the same extent as other species such as *Escherichia coli* or *Klebsiella* spp. Indeed, epidemiological relevance is an important factor supporting research priorities. According to the 2019 global mortality report [41], *E. coli* and *K. pneumoniae* are among the top four, being the most relevant representatives of the *Enterobacteriaceae* family. *Enterobacter* is included as a genus (*Enterobacter* spp.) within the top ten taxa [41]. Within the *Enterobacter* genus, *E. hormaechei* is an important pathogen, particularly relevant in healthcare-associated infections and for the emergence of antibiotic-resistant clones [42,43,44,45]. In this work, one of the four strains, identified as a type 3 fimbria-producing *E. hormaechei* subsp. *hoffmannii,* Eh31-UCH, was isolated from a sepsis case. It showed the capacity to resist multiple antibiotics, including a third-generation cephalosporin, likely by the production of ESBLs. We believe that research to gain knowledge into the pathogenic mechanisms and to identify colonization and virulence determinants is valuable for both antibiotic-susceptible and antibiotic-resistant strains. However, particularly for those strains displaying multiple resistance, this could help in exploring and developing novel therapies as alternatives to antibiotics. In this scenario, several anti-virulence, including anti-adherence, therapies have been proposed for *Enterobacteriaceae* representatives, as compounds to inhibit the production of attachment determinants, receptor analogs, probiotics, or vaccines, among others [9,46,47,48].

The type 3 fimbria was first described in *Klebsiella* spp., and most of the progress regarding knowledge about the structure has been made in species representative of that genus. Thus, the fimbria has even been proposed as a basis for potential anti-adherence therapies [13,14]. However, the presence of the type 3 fimbriae has been reported in other species as *E. coli*, *C. freundii*, and *C. koseri* [15]. In fact, an early work reported the reactivity of anti-type 3 fimbria with structures produced by several other representatives of the *Enterobacteriaceae* family, including some *Enterobacter* species [16]. Later, the presence of the *mrkB* gene was reported in *E. hormaechei* subsp. *oharae* isolates obtained in Brazil [18]. As higher BSR values were obtained in our screenings of the *mrkABCDF* locus, and also of the individual genes, sequences seem to be conserved between *K. pneumoniae* Kp13 and the positive *E. hormaechei* strains analyzed here. This observation is consistent with results reported by Ong et al., which indicated the high degree of conservation of the *mrkABCDF* locus among all the *Enterobacteriaceae* species in which the type 3 fimbria was detected (*Klebsiella* spp., *E. coli*, *C. freundii*, and *C. koseri*) [15].

Regarding the role of the type 3 fimbriae in the adherence capacity of *E. hormaechei* subsp. *hoffmannii*, we found that the Eh13-UCH mutant strain lacking *mrkA* had a significantly lower adherence capacity to a plastic plate compared to the wild type. The difference was particularly evident when the assay was performed in DMEM rather than LB, suggesting that the production of the type 3 fimbria could be favored in this medium. Previous data of higher production of the bundle-forming pilus and higher secretion of effector proteins by enteropathogenic *E. coli* cultured in DMEM, compared to LB, support this hypothesis [49,50]. Also, in agreement with this finding, the Eh13-UCH mutant strain lacking *mrkA* had a significantly lower adherence capacity to the human colonic epithelial cell line Caco-2, an assay that is also performed in DMEM. In both cases, adherence to the plastic surface and Caco-2 cells, and the complementation of the mutant strain with the *mrkA* gene, provided in the pVB1 expression plasmid, restored the adherent phenotype. This was evident at 3 h and 48 h over the plastic surface, in DMEM, and at 3 h over Caco-2 cells. Given that the cell-adherent phenotype of the complemented strain was not clear at 30 min, we hypothesize that type 3 fimbria production and/or assembly could take longer in that case. Although the colonic epithelium represents a reservoir rather than the most common *E. hormaechei* infection sites [1], the Caco-2 cell line has been previously used as a model to evaluate the adherence capacity of this bacterium [40]. In fact, according to the results reported by Rafferty et al., the number of CFU associated with the Caco-2 cells was higher than the number of CFU attached to primary human aortic endothelial cells [40]. This suggests that Caco-2 cells produce receptors for binding of the type 3 fimbria. If toxigenic or cytopathic effects occur over Caco-2 cells on *E. hormaechei* subsp. *hoffmannii* infection, and whether those are influenced by type 3 fimbria-directed binding, is yet to be determined. However, type 3 fimbria is conserved among *Enterobacteriaceae* species in which it has been detected, and it is involved in the adherence of *K. pneumoniae* to bladder and endothelial cells [51]. In addition, a role in the in vivo *K. pneumoniae* colonization of lungs and bladder in the mouse model has been reported [14,52]. Therefore, we hypothesize that it might also determine *E. hormaechei* attachment to other organs in which it causes infection.

According to our results, the overall presence of the type 3 fimbria among *E. hormaechei* is low. The highest percentages of genomes positive for *mrkABCDF* or *mrkC* in our study were observed among *E. hormaechei* subsp. *hoffmannii* (14/17%) and *E. hormaechei* subsp. *hormaechei* (17%) genomes. Even lower percentages were found among *E. hormaechei* subsp. *oharae*, *E. hormaechei* subsp. *steigerwaltii*, and *E. hormaechei* subsp. *xiangfangensis.* We performed the screening first with the *mrkABCDF* locus in order to find genomes harboring the minimum required number of genes to direct production of the type 3 fimbria, in a single piece of DNA. However, we also performed the screening of *mrkC* because the *mrkABCDF* might be incomplete in draft genomes, which represent most of the NCBI Assembly RefSeq database, due to incomplete sequencing. This is consistent with previous reports, in which type 3 fimbria-encoding genes have been detected among *E. cloacae* complex strains or *E. hormaechei* strains [17,18]. To our knowledge, there is only one report in which the presence of a type 3 fimbria-encoding gene was found in an *E. hormaechei* subspecies [18]. It corresponds to the *mrkB* gene detected in subpopulations of an *Enterobacter hormaechei* subsp. *oharae* displaying higher adherence capacities [18]. The presence of the *mrkABCDF* locus in plasmids, in most of the cases that we analyzed, suggests that it was acquired by representatives of several different lineages by horizontal transfer, as we found it in more than 50 different STs. The higher percentage of positivity for *E. hormaechei* subsp. *hoffmannii* and *E. hormaechei* subsp. *hormaechei* suggests that representatives of these subspecies could have experienced more encounters with type 3 fimbria-producing bacteria or that they have been more exposed to the free type 3 fimbria-encoding plasmids during their evolution. Alternatively, they might incorporate and/or deliver the type 3 fimbria-encoding plasmids more efficiently compared to representatives of the rest of the subspecies.

Even though there is a low distribution of type 3 fimbria among *E. hormaechei*, our results suggest that it might be a determinant of the colonization and pathogenic capacities in strains that have acquired the locus. This is consistent with the well-known role of the fimbria in *K. pneumoniae* and also with the fact that the most common *mrkABCDF-* and *mrkC*-positive sequence types found here have been found in carbapenem-resistant strains associated with illness in humans. For example, *E. cloacae* complex strains belonging to sequence types ST145, ST118, ST78, ST168, and ST66 were found in France, while ST145, ST93, and ST171 were found in the same type of strains isolated in China, and ST93 in Poland [53,54,55]. Furthermore, as bacterial adherence is a complex process, additional adhesive structures are expected to be produced by *E. hormaechei*, and future research will help to find and characterize them.

On the other hand, the characterization of *E. hormaechei* isolates at the subspecies level reinforces the relevance of the group, especially in nosocomial infections. For example, *E. hormaechei* subsp. *hoffmannii* was found as the most common agent of nosocomial infections in a University Hospital in Taiwan [56], and one of the agents was involved in an outbreak in a neonatal intensive care unit in Germany [57]. In addition, *E. hormaechei* strains, including *E. hormaechei* subsp. *xiangfangensis*, *E. hormaechei* subsp. *steigerwaltti,* and *E. hormaechei* subsp. *hoffmannii*, were found as the most common agents among *E. cloacae* complex isolates from hospitals in South Korea [58]. In that study, *E. hormaechei* subsp. *hoffmannii* and *E. hormaechei* subsp. *xiangfangensis* strains belonging to sequence types ST78 and ST66, respectively, both recognized in our study for including *mrkABCDF*-positive representatives, were identified [58].

Given that the taxonomy of *Enterobacter* spp. has been evolving, the identity of the isolates at the species and/or subspecies level has not always been established according to a single scheme. In 2020, Wu et al. proposed an update on the taxonomy of the *Enterobacter* genus based on comparative genomics and phylogenomic analyses [5]. In that work, *E. hormaechei* subsp. *hoffmannii* was proposed to be renamed as *E. hoffmannii*; so, the Eh12-UCH, Eh13-UCH, Eh18-UCH, and Eh31-UCH strains could also be considered as *E. hoffmannii*. In addition, *E. hormaechei* subsp. *oharae* and *E. hormaechei* subsp. *steigerwaltii* were considered as synonyms of *E. xiangfangensis*. Therefore, several database records identified here as positive for *mrkABCDF* or *mrkC*, indexed as *E. hormaechei* subsp. *oharae* or *E. hormaechei* subsp. *steigerwaltii*, might represent *E. xiangfangensis*. This proposal remains to be considered in the List of Prokaryotic Names with Standing in Nomenclature [4]. However, some studies have already incorporated the new guidelines and have identified these species. For example, a study carried out in France found *E. hoffmannii*, *E. hormaechei*, *and E. xiangfangensis* among strains obtained from septic shock lethal cases in newborns [59]. Another study reanalyzed the presence of AmpC variants with *E. hoffmannii* and *E. xiangfangensis* strains isolated from different clinical samples in China [60]. A third study carried out in France, found *E. hoffmanni* and *E. xiangfangensis* among strains causing neonatal sepsis cases, attributed to contamination of the incubators [61]. Finally, a study carried out in Spain identified *E. hoffmannii* strains belonging to the ST78 sequence type and *E. xiangfangensis* strains belonging to the ST66 and ST171 sequence types as causative agents of bloodstream infections [62]. On the other hand, a very recent study in which genomes of 256 clinical strains belonging to the *E. cloacae* complex were analyzed suggests that the subspecies classification scheme for *E. hormaechei* has a better correlation with the molecular features, including phylogeny, presence of virulence genes, and capsule type [63]. Indeed, regardless of this dynamic scenario, the current epidemiological relevance of the *Enterobacter* species is evident in the results of all these studies.

Overall, it is expected that the knowledge about *Enterobacter* species will move forward regarding taxonomy and phylogenomics. These advances should certainly move along with increasing knowledge regarding pathogenicity mechanisms and the role and distribution of the key virulence factors.

## 5. Conclusions

1. The type 3 fimbria is an adherence determinant of *Enterobacter hormaechei* subsp. *hoffmannii*.
2. The type 3 fimbria is uncommon among *E. hormaechei*, although it can be harbored by representatives of the five subspecies. *E. hormaechei* subsp. *hoffmannii* and *E. hormaechei* subsp. *hormaechei* displayed the highest positivity rates. Production of type 3 fimbria may confer fitness advantages regarding adherence and colonization capacities.

## Figures and Tables

**Table 1:** Strains and plasmids used in this work.

Strain	Specie/Complex	Origin/Illness	Utility	Reference
Eclo1-UCH	*Enterobacter cloacae*	Pneumonia	Detection of type 3 fimbria	This study
Eclo5-UCH	*Enterobacter cloacae*	Sepsis	Detection of type 3 fimbria	This study
Eclo6-UCH	*Enterobacter cloacae*	Sepsis	Detection of type 3 fimbria	This study
Eh12-UCH	*Enterobacter hormaechei* subsp. *hoffmannii*	Sepsis	Detection of type 3 fimbria	This study
Eh13-UCH	*Enterobacter hormaechei* subsp. *hoffmannii*	Sepsis	Detection of type 3 fimbria and functional analyses	This study
Eh18-UCH	*Enterobacter hormaechei* subsp. *hoffmannii*	Pneumonia	Detection of type 3 fimbria	This study
Eclo29-UCH	*Enterobacter cloacae*	Sepsis	Detection of type 3 fimbria	This study
Eh31-UCH	*Enterobacter hormaechei* subsp. *hoffmannii*	Sepsis	Detection of type 3 fimbria	This study
Kpn1-UCH	*Klebsiella pneumoniae*	Pneumonia	Detection of type 3 fimbria	This study
Eh13-UCHΔ*mrkA*	*Enterobacter hormaechei* subsp. *hoffmannii*	Obtained after removal of the *mrkA* gene in Eh13-UCH	Detection of type 3 fimbria	This study
Eh13-UCHΔ*mrkA/*pVB1	*Enterobacter hormaechei* subsp. *hoffmannii*	Eh13-UCHΔ*mrkA* transformed with the empty pVB1 plasmid	Detection of type 3 fimbria	This study
Eh13-UCHΔ*mrkA/mrkA*	*Enterobacter hormaechei* subsp. *hoffmannii*	Eh13-UCHΔ*mrkA* transformed with the pVB1 plasmid containing *mrkA*	Detection of type 3 fimbria	This study
Plasmids				
pCLF4	-	-	Template to amplify the kanamycin resistance cassette used for allelic exchange	[20]
pSIM9	-	-	Provision of the λ Red recombinase system	[21]
pVB1	-	-	Expression plasmid, promoter inducible with m-tuolic acid	DualSystems Biotech, (Schlieren, Switzerland)

**Table 2:** Main features of the draft genomes obtained in this study.

Strain	Sequence Type	Genome Length (bp)	N50	Completeness (%)	Contamination (%)	NCBI Nucleotide Accession Code
Eh12-UCH	ST145	5,115,276	270,304	99.89	0.2	JBCNUO000000000
Eh13-UCH	ST145	4,744,088	309,640	99.89	0.2	JBCNUP000000000
Eh18-UCH	ST145	5,112,462	269,969	99.89	0.2	JBCNUQ000000000
Eh31-UCH	ST118	5,900,299	165,594	99.78	12.04	JBCNUR000000000

## Data Availability

The original contributions presented in the study are included in the article and Supplementary Materials, further inquiries can be directed to the corresponding author.

## Supplementary Materials

The following supporting information can be downloaded at: https://www.mdpi.com/article/10.3390/microorganisms12071441/s1, Table S1: Primers used in this work; Table S2: Sequences recovered from databases, used in this study. Table S3: Antibiotic resistance genes identified in *E. hormaechei* subsp. *hoffmannii* sequenced in this study and their known resistance-associated profiles according to ResFinder. Reference [64] is cited in the Supplementary Materials.

## References

[1] Davin-Regli A., Lavigne J.P., Pagès J.M. (2019). *Enterobacter* spp.: Update on taxonomy, clinical aspects, and emerging antimicrobial resistance. Clin. Microbiol. Rev..

[2] Martins E.R., Bueno M.F.C., Francisco G.R., Casella T., de Oliveira Garcia D., Cerdeira L.T., Gerber A.L., de Almeida L.G.P., Lincopan N., de Vasconcelos A.T.R. (2020). Genome and plasmid context of two *rmtG*-carrying *Enterobacter hormaechei* isolated from urinary tract infections in Brazil. J. Glob. Antimicrob. Resist..

[3] da Silva C.L., Miranda L.E., Moreira B.M., Rebello D., Carson L.A., Kellum M.E., de Almeida M.C., Sampaio J.L., O’Hara C.M. (2002). *Enterobacter hormaechei* bloodstream infection at three neonatal intensive care units in Brazil. Pediatr. Infect. Dis. J..

[4] Parte A.C., Sardà Carbasse J., Meier-Kolthoff J.P., Reimer L.C., Göker M. (2020). List of prokaryotic names with standing in nomenclature (LPSN) moves to the DSMZ. Int. J. Syst. Evol. Microbiol..

[5] Wu W., Feng Y., Zong Z. (2020). Precise species identification for *Enterobacter*: A genome sequence-based study with reporting of two novel species, *Enterobacter quasiroggenkampii* sp. nov. and *Enterobacter quasimori* sp. nov. mSystems.

[6] Rice L.B. (2008). Federal funding for the study of antimicrobial resistance in nosocomial pathogens: No ESKAPE. J. Infect. Dis..

[7] De Rosa F.G., Corcione S., Pagani N., Di Perri G. (2015). From ESKAPE to ESCAPE, from KPC to CCC. Clin. Infect. Dis..

[8] WHO Bacterial Priority Pathogens List, 2024: Bacterial Pathogens of Public Health Importance to Guide Research, Development and Strategies to Prevent and Control Antimicrobial Resistance. https://www.who.int/publications/i/item/9789240093461.

[9] Campos J.C.M., Antunes L.C., Ferreira R.B. (2020). Global priority pathogens: Virulence, antimicrobial resistance and prospective treatment options. Future Microbiol..

[10] Gerlach G.F., Allen B.L., Clegg S. (1988). Molecular characterization of the type 3 (MR/K) fimbriae of *Klebsiella pneumoniae*. J. Bacteriol..

[11] Nuccio S.P., Bäumler A.J. (2007). Evolution of the chaperone/usher assembly pathway: Fimbrial classification goes Greek. Microbiol. Mol. Biol. Rev..

[12] Murphy C.N., Clegg S. (2012). *Klebsiella pneumoniae* and type 3 fimbriae: Nosocomial infection, regulation and biofilm formation. Future Microbiol..

[13] Choi M., Tennant S.M., Simon R., Cross A.S. (2019). Progress towards the development of *Klebsiella* vaccines. Expert. Rev. Vaccines.

[14] Wang Q., Chang C.S., Pennini M., Pelletier M., Rajan S., Zha J., Chen Y., Cvitkovic R., Sadowska A., Heidbrink Thompson J. (2016). Target-agnostic identification of functional monoclonal antibodies against *Klebsiella pneumoniae* multimeric MrkA fimbrial subunit. J. Infect. Dis..

[15] Ong C.L., Beatson S.A., Totsika M., Forestier C., McEwan A.G., Schembri M.A. (2010). Molecular analysis of type 3 fimbrial genes from *Escherichia coli*, *Klebsiella* and *Citrobacter* species. BMC Microbiol..

[16] Old D.C., Adegbola R.A. (1985). Antigenic relationships among type-3 fimbriae of *Enterobacteriaceae* revealed by immunoelectronmicroscopy. J. Med. Microbiol..

[17] Livrelli V., De Champs C., Di Martino P., Darfeuille-Michaud A., Forestier C., Joly B. (1996). Adhesive properties and antibiotic resistance of *Klebsiella*, *Enterobacter*, and *Serratia* clinical isolates involved in nosocomial infections. J. Clin. Microbiol..

[18] Brust F.R., Boff L., da Silva Trentin D., Pedrotti Rozales F., Barth A.L., Macedo A.J. (2019). Macrocolony of NDM-1 producing *Enterobacter hormaechei* subsp. *oharae* generates subpopulations with different features regarding the response of antimicrobial agents and biofilm formation. Pathogens.

[19] Gales A.C., Castanheira M., Jones R.N., Sader H.S. (2012). Antimicrobial resistance among Gram-negative bacilli isolated from Latin America: Results from SENTRY Antimicrobial Surveillance Program (Latin America, 2008–2010). Diagn. Microbiol. Infect. Dis..

[20] Santiviago C.A., Reynolds M.M., Porwollik S., Choi S.H., Long F., Andrews-Polymenis H.L., McClelland M. (2009). Analysis of pools of targeted *Salmonella* deletion mutants identifies novel genes affecting fitness during competitive infection in mice. PLoS Pathog..

[21] Sharan S.K., Thomason L.C., Kuznetsov S.G., Court D.L. (2009). Recombineering: A homologous recombination-based method of genetic engineering. Nat. Protoc..

[22] *Performance Standards for Antimicrobial Susceptibility Testing, M100*, 32nd ed.; Clinical and Laboratory Standards Institute: Wayne, PA, USA, 2022. https://clsi.org/standards/products/microbiology/documents/m100/.

[23] Prjibelski A., Antipov D., Meleshko D., Lapidus A., Korobeynikov A. (2020). Using SPAdes De Novo Assembler. Curr. Protoc. Bioinform..

[24] Gurevich A., Saveliev V., Vyahhi N., Tesler G. (2013). QUAST: Quality assessment tool for genome assemblies. Bioinformatics.

[25] Parks D.H., Imelfort M., Skennerton C.T., Hugenholtz P., Tyson G.W. (2015). CheckM: Assessing the quality of microbial genomes recovered from isolates, single cells, and metagenomes. Genome Res..

[26] Jolley K.A., Bliss C.M., Bennett J.S., Bratcher H.B., Brehony C., Colles F.M., Wimalarathna H., Harrison O.B., Sheppard S.K., Cody A.J. (2012). Ribosomal multilocus sequence typing: Universal characterization of bacteria from domain to strain. Microbiology.

[27] Gardner S.N., Slezak T., Hall B.G. (2015). kSNP3.0: SNP detection and phylogenetic analysis of genomes without genome alignment or reference genome. Bioinformatics.

[28] Jain C., Rodriguez-R L.M., Phillippy A.M., Konstantinidis K.T., Aluru S. (2018). High throughput ANI analysis of 90K prokaryotic genomes reveals clear species boundaries. Nat. Commun..

[29] Seemann T. mlst. Github. https://github.com/tseemann/mlst.

[30] Jolley K.A., Bray J.E., Maiden M.C.J. (2018). Open-access bacterial population genomics: BIGSdb software, the PubMLST.org website and their applications. Wellcome Open Res..

[31] Bortolaia V., Kaas R.S., Ruppe E., Roberts M.C., Schwarz S., Cattoir V., Philippon A., Allesoe R.L., Rebelo A.R., Florensa A.F. (2020). ResFinder 4.0 for predictions of phenotypes from genotypes. J. Antimicrob. Chemother..

[32] Camacho C., Coulouris G., Avagyan V., Ma N., Papadopoulos J., Bealer K., Madden T.L. (2009). BLAST+: Architecture and applications. BMC Bioinform..

[33] Ramos P.I., Picão R.C., Almeida L.G., Lima N.C., Girardello R., Vivan A.C., Xavier D.E., Barcellos F.G., Pelisson M., Vespero E.C. (2014). Comparative analysis of the complete genome of KPC-2-producing *Klebsiella pneumoniae* Kp13 reveals remarkable genome plasticity and a wide repertoire of virulence and resistance mechanisms. BMC Genom..

[34] Sayers E.W., Bolton E.E., Brister J.R., Canese K., Chan J., Comeau D.C., Connor R., Funk K., Kelly C., Kim S. (2022). Database resources of the national center for biotechnology information. Nucleic Acids Res..

[35] Fernández-Yáñez V., Suazo P., Hormazábal C., Ibaceta V., Arenas-Salinas M., Vidal R.M., Silva-Ojeda F., Arellano C., Muñoz I., Del Canto F. (2024). Distribution of *papA* and *papG* variants among *Escherichia coli* genotypes: Association with major extraintestinal pathogenic lineages. Int. J. Mol. Sci..

[36] Wang L., Wu P., Su Y., Wei Y., Guo X., Yang L., Wang M., Liu B. (2022). Detection of genus and three important species of *Cronobacter* using novel genus- and species-specific genes identified by large-scale comparative genomic analysis. Front. Microbiol..

[37] Webb J.R., Buller N., Rachlin A., Golledge C., Sarovich D.S., Price E.P., Mayo M., Currie B.J. (2020). A persisting nontropical focus of *Burkholderia pseudomallei* with limited genome evolution over five decades. mSystems.

[38] Del Canto F., Botkin D.J., Valenzuela P., Popov V., Ruiz-Perez F., Nataro J.P., Levine M.M., Stine O.C., Pop M., Torres A.G. (2012). Identification of Coli Surface Antigen 23, a novel adhesin of enterotoxigenic *Escherichia coli*. Infect. Immun..

[39] Bradford M.M. (1976). A rapid and sensitive method for the quantitation of microgram quantities of protein utilizing the principle of protein-dye binding. Anal. Biochem..

[40] Rafferty B., Dolgilevich S., Kalachikov S., Morozova I., Ju J., Whittier S., Nowygrod R., Kozarov E. (2011). Cultivation of *Enterobacter hormaechei* from human atherosclerotic tissue. J. Atheroscler. Thromb..

[41] GBD 2019 Antimicrobial Resistance Collaborators (2022). Global mortality associated with 33 bacterial pathogens in 2019: A systematic analysis for the Global Burden of Disease Study 2019. Lancet.

[42] Yeh T.K., Lin H.J., Liu P.Y., Wang J.H., Hsueh P.R. (2022). Antibiotic resistance in *Enterobacter hormaechei*. Int. J. Antimicrob. Agents.

[43] Xu T., Xue C.X., Huang J., Wu J., Chen R., Zhou K. (2022). Emergence of an epidemic hypervirulent clone of *Enterobacter hormaechei* coproducing *mcr-9* and carbapenemases. Lancet Microbe.

[44] Knecht C.A., García Allende N., Álvarez V.E., Prack Mc Cormick B., Massó M.G., Campos J., Fox B., Alonso F.M., Donis N., Canigia L.F. (2022). New sequence type of an *Enterobacter cloacae* complex strain with the potential to become a high-risk clone. J. Glob. Antimicrob. Resist..

[45] Donà V., Nordmann P., Kittl S., Schuller S., Bouvier M., Poirel L., Endimiani A., Perreten V. (2023). Emergence of OXA-48-producing *Enterobacter hormaechei* in a Swiss companion animal clinic and their genetic relationship to clinical human isolates. J. Antimicrob. Chemother..

[46] Cegelski L., Marshall G.R., Eldridge G.R., Hultgren S.J. (2008). The biology and future prospects of antivirulence therapies. Nat. Rev. Microbiol..

[47] Krachler A.M., Orth K. (2013). Targeting the bacteria-host interface: Strategies in anti-adhesion therapy. Virulence.

[48] Lau W.Y.V., Taylor P.K., Brinkman F.S.L., Lee A.H.Y. (2023). Pathogen-associated gene discovery workflows for novel antivirulence therapeutic development. eBioMedicine.

[49] Puente J.L., Bieber D., Ramer S.W., Murray W., Schoolnik G.K. (1996). The bundle-forming pili of enteropathogenic *Escherichia coli*: Transcriptional regulation by environmental signals. Mol. Microbiol..

[50] Kenny B., Abe A., Stein M., Finlay B.B. (1997). Enteropathogenic *Escherichia coli* protein secretion is induced in response to conditions similar to those in the gastrointestinal tract. Infect. Immun..

[51] Tarkkanen A.M., Virkola R., Clegg S., Korhonen T.K. (1997). Binding of the type 3 fimbriae of *Klebsiella pneumoniae* to human endothelial and urinary bladder cells. Infect. Immun..

[52] Murphy C.N., Mortensen M.S., Krogfelt K.A., Clegg S. (2013). Role of *Klebsiella pneumoniae* type 1 and type 3 fimbriae in colonizing silicone tubes implanted into the bladders of mice as a model of catheter-associated urinary tract infections. Infect. Immun..

[53] Emeraud C., Petit C., Gauthier L., Bonnin R.A., Naas T., Dortet L. (2022). Emergence of VIM-producing *Enterobacter cloacae* complex in France between 2015 and 2018. J. Antimicrob. Chemother..

[54] Chen J., Tian S., Nian H., Wang R., Li F., Jiang N., Chu Y. (2021). Carbapenem-resistant *Enterobacter cloacae* complex in a tertiary Hospital in Northeast China, 2010–2019. BMC Infect. Dis..

[55] Izdebski R., Baraniak A., Zabicka D., Sekowska A., Gospodarek-Komkowska E., Hryniewicz W., Gniadkowski M. (2018). VIM/IMP carbapenemase-producing *Enterobacteriaceae* in Poland: Epidemic *Enterobacter hormaechei* and *Klebsiella oxytoca* lineages. J. Antimicrob. Chemother..

[56] Chen C.J., Lu P.L., Jian S.H., Fu H.L., Huang P.H., Chang C.Y. (2022). Molecular epidemiology, risk factors and clinical outcomes of carbapenem-nonsusceptible *Enterobacter cloacae* complex infections in a Taiwan University Hospital. Pathogens.

[57] Morhart P., Gerlach R.G., Kunz C., Held J., Valenza G., Wölfle J., Reutter H., Hanslik G.J., Fahlbusch F.B. (2023). Application of next-generation sequencing to *Enterobacter hormaechei* subspecies analysis during a neonatal intensive care unit outbreak. Children.

[58] Ganbold M., Seo J., Wi Y.M., Kwon K.T., Ko K.S. (2023). Species identification, antibiotic resistance, and virulence in *Enterobacter cloacae* complex clinical isolates from South Korea. Front. Microbiol..

[59] Girlich D., Ouzani S., Emeraud C., Gauthier L., Bonnin R.A., Le Sache N., Mokhtari M., Langlois I., Begasse C., Arangia N. (2021). Uncovering the novel *Enterobacter cloacae* complex species responsible for septic shock deaths in newborns: A cohort study. Lancet Microbe.

[60] Feng Y., Hu Y., Zong Z. (2021). Reexamining the association of AmpC variants with *Enterobacter* species in the context of updated taxonomy. Antimicrob. Agents Chemother..

[61] Hernandez-Alonso E., Bourgeois-Nicolaos N., Lepainteur M., Derouin V., Barreault S., Waalkes A., Augusto L.A., Gera S., Gleizes O., Tissieres P. (2022). Contaminated incubators: Source of a multispecies *Enterobacter* outbreak of neonatal sepsis. Microbiol. Spectr..

[62] Lumbreras-Iglesias P., de Toro M., Vázquez X., García-Carús E., Rodicio M.R., Fernández J. (2023). High-risk international clones ST66, ST171 and ST78 of *Enterobacter cloacae* complex causing blood stream infections in Spain and carrying *blaOXA-48* with or without *mcr-9*. J. Infect. Public. Health.

[63] Qiu X., Ye K., Ma Y., Zhao Q., Wang L., Yang J. (2024). Genome sequence-based species classification of *Enterobacter cloacae* complex: A study among clinical isolates. Microbiol. Spectr..

[64] Datsenko K.A., Wanner B.L. (2000). One-step inactivation of chromosomal genes in *Escherichia coli* K-12 using PCR products. Proc. Natl. Acad. Sci. USA.

