# Supplementary material for: Presence and Role of the Type 3 Fimbria in the Adherence Capacity of Enterobacter hormaechei subsp. hoffmannii
Source: Microorganisms. 2024 Jul 16;12(7):1441. doi: 10.3390/microorganisms12071441 (PMC11279048; doi:10.3390/microorganisms12071441)
Supplement: Supplementary file 1 [file microorganisms-12-01441-s001.zip › microorganisms-3082041-supplementary.pdf]

Table S1. Primers used in this work

Primer	Sequence (5'-3')	Utility	Reference
mrkA-F	AGGTTAATTTCTTCGGTAAAGTTAC	Detection of *mrkA* gene	This study
mrkA-R	CGGGATGATTTTGTTGGTCAG	Detection of *mrkA* gene	This study
mrkA-mutF	TCTGCAGCAATGGCAACCGCGTTTTTGGCATGACTG CTGGTGTAGGCTGGAGCTGCTTC	Amplification of the kanamycin resistance cassette from pCFL4, in allelic exchange for removal of *mrkA* gene	This study
mrkA-mutR	TTACTGGTAAGTAATTTCGTAAGTCGCGTAGCTGTTA ACCCATATGAATATCCTCCTTAG	Amplification of the kanamycin resistance cassette from pCFL4, in allelic exchange for removal of *mrkA* gene	This study
mrkA-Nde	AGCCATATGAAAAAGGTTCTTCTCTCTGC	Amplification of *mrkA* gene for ligation onto pVB1 plasmid. Contains an NdeI restriction site.<br><br>Checking of the *mrkA* allelic replacement	This study
mrkA-Bam	ACGGATCCTTACTGGTAAGTAATTTCGTAAGTC	Amplification of *mrkA* gene for ligation onto pVB1 plasmid. Contains a BamHI restriction site	This study
K1	CAGTCATAGCCGAATAGCCT	Checking of the *mrkA* allelic replacement	[64]

Table S2. Sequences recovered from databases, used in this study.

Record	NCBI accession code (positions)	Utility
Klebsiella pneumoniae Kp13 *mrkABCDF* locus	Nucleotide CP003999.1 (842,841-848,385)	Screening of *mrkABCDF* locus
Klebsiella pneumoniae Kp13 *mrkA* gene	Nucleotide CP003999.1 (842,841-843,455)	Screening of *mrkA* gene
Klebsiella pneumoniae Kp13 *mrkB* gene	Nucleotide CP003999.1 (843,551-844,252)	Screening of *mrkB* gene
Klebsiella pneumoniae Kp13 *mrkC* gene	Nucleotide CP003999.1 (844,264-846,750)	Screening of *mrkC* gene
Klebsiella pneumoniae Kp13 *mrkD* gene	Nucleotide CP003999.1 (846,741-847,736)	Screening of *mrkD* gene
Klebsiella pneumoniae Kp13 *mrkF* gene	Nucleotide CP003999.1 (847,750-848,385)	Screening of *mrkF* gene
Enterobacter hormaechei subsp. *hoffmannii* strain DSM 14563	Assembly Refseq GCF_001729745	Subspecies assignations
Enterobacter hormaechei subsp. *hormaechei* ATCC 49162	Assembly Refseq GCF_001875655	Subspecies assignations
Enterobacter hormaechei subsp. *oharae* DSM 16687	Assembly Refseq GCF_001729705	Subspecies assignations
Enterobacter hormaechei subsp. *steigerwaltii* DSM 16691	Assembly Refseq GCF_001729725	Subspecies assignations
Enterobacter hormaechei subsp. *xiangfangensis* LMG27195	Assembly Refseq GCF_001729785.1	Subspecies assignations
Enterobacter hormaechei AR432	Assembly Refseq GCF_003073995.1	*mrkABCDF* locus localization
Enterobacter hormaechei L51	Assembly Refseq GCF_009738085.1	*mrkABCDF* locus localization
Enterobacter hormaechei RHBSTW-00114	Assembly Refseq GCF_013625115.1	*mrkABCDF* locus localization
Enterobacter hormaechei RHBSTW-00105	Assembly Refseq GCF_013625265.1	*mrkABCDF* locus localization
Enterobacter hormaechei RHBSTW-00083	Assembly Refseq GCF_013625265.1	*mrkABCDF* locus localization
Enterobacter hormaechei RHBSTW-00059	Assembly Refseq GCF_013629235.1	*mrkABCDF* locus localization

Enterobacter hormaechei	RHBSTW-00056	Assembly Refseq GCF_013629455.1	*mrkABCDF* locus localization
Enterobacter hormaechei	RHBSTW-00040	Assembly Refseq GCF_013629675.1	*mrkABCDF* locus localization
Enterobacter hormaechei	RHBSTW-00016	Assembly Refseq GCF_013635255.1	*mrkABCDF* locus localization
Enterobacter hormaechei	RHBSTW-00012	Assembly Refseq GCF_013635495.1	*mrkABCDF* locus localization
Enterobacter hormaechei	RHBSTW-00916	Assembly Refseq GCF_013726395.1	*mrkABCDF* locus localization
Enterobacter hormaechei	RHBSTW-00844	Assembly Refseq GCF_013731155.1	*mrkABCDF* locus localization
Enterobacter hormaechei	RHBSTW-00642	Assembly Refseq GCF_013734275.1	*mrkABCDF* locus localization
Enterobacter hormaechei	RHBSTW-00333	Assembly Refseq GCF_013738375.1	*mrkABCDF* locus localization
Enterobacter hormaechei	RHBSTW-00668	Assembly Refseq GCF_013739595.1	*mrkABCDF* locus localization
Enterobacter hormaechei	RHBSTW-00492	Assembly Refseq GCF_013740655.1	*mrkABCDF* locus localization
Enterobacter hormaechei	RHBSTW-00316	Assembly Refseq GCF_013742375.1	*mrkABCDF* locus localization
Enterobacter hormaechei	RHBSTW-00198	Assembly Refseq GCF_013744415.1	*mrkABCDF* locus localization
Enterobacter hormaechei	ENCL48880	Assembly Refseq GCF_013873515.1	*mrkABCDF* locus localization
Enterobacter hormaechei	BD-50-Eh	Assembly Refseq GCF_015535675.1	*mrkABCDF* locus localization
Enterobacter hormaechei	K528	Assembly Refseq GCF_023375605.1	*mrkABCDF* locus localization
Enterobacter hormaechei	4453	Assembly Refseq GCF_024917635.1	*mrkABCDF* locus localization
Enterobacter hormaechei	RH3	Assembly Refseq GCF_037217235.1	*mrkABCDF* locus localization

Table S3. Antibiotic resistance genes identified in *E. hormaechei* subsp. *hoffmannii* sequenced in this study and their known resistance-associated profiles according to ResFinder.

Strain	Antibiotic resistance genes (% identity)	Predicted resistance profile*
**Eh12-UCH**	$bla_{\text{ACT-14}}$ (98.69%)	Amoxicillin<br>Amoxicillin + clavulanic acid<br>**Ampicillin**<br>Ampicillin + clavulanic acid<br>Cefotaxime<br>Cefoxitin<br>Ceftazidime<br>Piperacillin<br>**Piperacillin + tazobactam**<br>Ticarcillin<br>Ticarcillin + clavulanic acid
	fosA (96.01%)	Fosfomycin
**Eh13-UCH**	$bla_{\text{ACT-14}}$ (98.78%)	Amoxicillin<br>Amoxicillin + clavulanic acid<br>**Ampicillin**<br>Ampicillin + clavulanic acid<br>Cefotaxime<br>Cefoxitin<br>Ceftazidime<br>Piperacillin<br>**Piperacillin + tazobactam**<br>Ticarcillin<br>Ticarcillin + clavulanic acid
	fosA (96.01%)	Fosfomycin
**Eh18-UCH**	$bla_{\text{ACT-14}}$ (98.69%)	Amoxicillin<br>Amoxicillin + clavulanic acid<br>**Ampicillin**<br>Ampicillin + clavulanic acid<br>Cefotaxime<br>Cefoxitin<br>Ceftazidime<br>Piperacillin<br>**Piperacillin + tazobactam**<br>Ticarcillin<br>Ticarcillin + clavulanic acid

	fosA (96.01%)	Fosfomycin
**Eh31-UCH**	*aph(6)-ld* / *strB* (100%)	**Streptomycin**
	aac(6′)-llc (100%)	**Gentamycin**<br>Tobramycin
	aph(3)-la (100%)	**Aminoglycosides**
	aac(6′)-lb3	Amikacin<br>Tobramycin
	bla$_{ACT-14}$ / *bla*$_{ACT-5}$ (98.78%)	Amoxicillin<br>Amoxicillin + clavulanic acid<br>**Ampicillin**<br>Ampicillin + clavulanic acid<br>**Cefotaxime**<br>Cefoxitin<br>Ceftazidime<br>Piperacillin<br>Piperacillin + tazobactam<br>Ticarcillin<br>Ticarcillin + clavulanic acid
	bla$_{TEM-1B}$ (100%)	Amoxicillin<br>Ampicillin<br>Piperacillin<br>Ticarcillin<br>**Cephalothin**
	bla$_{DHA-1}$ (100%)	Amoxicillin<br>Amoxicillin + clavulanic acid<br>**Ampicillin**<br>Ampicillin + clavulanic acid<br>**Cefotaxime**<br>Cefoxitin<br>Ceftazidime<br>Piperacillin<br>**Piperacillin + tazobactam**<br>Ticarcillin<br>Ticarcillin + clavulanic acid
	bla$_{SHV-12}$ (100%)	Amoxicillin<br>**Ampicillin**<br>Cefepime

		**Cefotaxime**<br>Ceftazidime<br>Piperacillin<br>Aztreonam<br>Ticarcillin<br>Ceftriaxone
	fosA (98.84%)	Fosfomycin
	ere(A)	Erythromycin
	catA2	**Chloramphenicol**
	qnrB4	Ciprofloxacin
	sul1	Sulfamethoxazole
	tet(D)	**Tetracycline**<br>Doxycycline
	dfrA19	**Trimethoprim**

*phenotypes observed in disk-diffusion assays are written in bold letter.
